# Fecal Nervonic Acid as a Biomarker for Diagnosing and Monitoring Inflammatory Bowel Disease

**DOI:** 10.3390/biomedicines12122764

**Published:** 2024-12-04

**Authors:** Claudia Kunst, Tanja Elger, Johanna Loibl, Muriel Huss, Arne Kandulski, Sabrina Krautbauer, Martina Müller, Gerhard Liebisch, Hauke Christian Tews, Christa Buechler

**Affiliations:** 1 Department of Internal Medicine I, Gastroenterology, Hepatology, Endocrinology, Rheumatology, and Infectious Diseases, University Hospital Regensburg, 93053 Regensburg, Germany; claudia.kunst@klinik.uni-regensburg.de (C.K.); tanja.elger@klinik.uni-regensburg.de (T.E.); johanna.loibl@klinik.uni-regensburg.de (J.L.); muriel.huss@klinik.uni-regensburg.de (M.H.);arne.kandulski@klinik.uni-regensburg.de (A.K.); martina.mueller-schilling@klinik.uni-regensburg.de (M.M.); hauke.tews@klinik.uni-regensburg.de (H.C.T.); 2 Institute of Clinical Chemistry and Laboratory Medicine, University Hospital Regensburg, 93053 Regensburg, Germany; sabrina.krautbauer@klinik.uni-regensburg.de (S.K.); gerhard.liebisch@klinik.uni-regensburg.de (G.L.)

**Keywords:** nervonic acid, fatty acids, biomarker, ulcerative colitis, Crohn’s disease, inflammatory bowel disease

## Abstract

Background/Objectives: Inflammatory bowel disease (IBD) is a chronic immune-mediated pathology associated with the dysregulation of lipid metabolism. The administration of nervonic acid, a very long-chain fatty acid, has been shown to improve colonic inflammation in a mouse model of colitis. Our study aimed to quantify fecal levels of nervonic acid, as well as the very long-chain fatty acids, lignoceric acid, and pentacosanoic acid, to identify associations with IBD activity. Methods: Stool samples were collected from 62 patients with IBD and 17 healthy controls. Nervonic acid, lignoceric acid, and pentacosanoic acid were quantified by gas chromatography coupled with mass spectrometry (GC-MS). Lipid levels, normalized to the dry weight of fecal homogenates, were used for calculations. Results: Patients with IBD exhibited elevated fecal nervonic acid levels compared to healthy controls, with no significant differences observed between ulcerative colitis and Crohn’s disease. A fecal nervonic acid concentration of 0.49 µmol/g distinguished IBD patients from controls, achieving a sensitivity of 71% and a specificity of 82%. Fecal nervonic acid levels showed a positive correlation with both C-reactive protein and fecal calprotectin and increased proportionally with rising fecal calprotectin levels. IBD patients treated with corticosteroids or interleukin-12/23 antibodies had higher levels of fecal nervonic acid than those in other therapies, with no difference in serum C-reactive protein and calprotectin levels between these groups. Conclusions: In summary, this analysis indicates that fecal nervonic acid may emerge as a novel specific biomarker for IBD diagnosis and disease monitoring.

## 1. Introduction

Inflammatory bowel diseases (IBD), including the main forms of Crohn’s disease (CD) and ulcerative colitis (UC), are chronic inflammatory diseases of the gastrointestinal tract [1,2]. Due to the early onset and chronicity of these diseases, their global prevalence is projected to increase to about 1% over the next few decades [3]. The precise pathomechanisms of these multifactorial diseases are still unclear. It is hypothesized that an environmental trigger initiates an abnormal immune response in the intestines of genetically predisposed individuals [4,5,6]. Thus, the immunological homeostasis within the intestinal mucosa is disturbed by self-perpetuating inflammatory processes. Maintained inflammation is associated with increased cell death rates in biopsy samples from patients with CD and UC as well as in mouse models of colitis [7,8,9,10,11].

Fatty acids have a variety of roles in IBD, from immunoregulatory responses to the modulation of barrier integrity. The gut microbiota ferments indigestible dietary fiber to produce short-chain fatty acids, which have potent anti-inflammatory properties and are thought to be important protective factors against IBD. Polyunsaturated fatty acids mostly have anti-inflammatory properties, whereas saturated fatty acids have pro-inflammatory effects [12,13,14,15]. Dietary supplementation with the long-chain polyunsaturated fatty acid, eicosapentaenoic acid, for six months, reduced fecal calprotectin levels without causing significant side effects in a placebo-controlled trial in patients with UC [16].

Very long-chain fatty acids (VLCFAs) are fatty acids with more than 22 carbon atoms in their structure. VLCFAs have essential biological functions, including the formation of cellular membranes, signaling processes, and the maintenance of skin barrier integrity. In addition to being produced endogenously from long-chain fatty acids and elongated by the elongase of the very long fatty acids family, VLCFAs can also be obtained from food [15,17]. Linked to a sphingoid base, VLCFAs are important structural components of sphingolipids [18].

Nervonic acid is a monounsaturated VLCFA primarily found in the white matter of animal brains and in high concentrations in the human liver and kidney. Nervonic acid contributes to metabolic health, regulates the immune system, and possesses anti-inflammatory properties [19]. Moreover, recent research indicated that diets supplemented with nervonic acid not only exert positive effects on human health in general, but also have the potential to improve various medical conditions, including neurological diseases, cancer, diabetes, obesity, and associated complications [19,20,21,22].

Oral administration of nervonic acid in a mouse model of colitis demonstrated anti-inflammatory properties by suppressing NF-κB-mediated signaling and restoring intestinal barrier function [22]. Moreover, nervonic acid-dependent reduction in epithelial damage and pro-inflammatory cytokine production in the colon of mice with colitis was comparable to those of eicosapentaenoic acid and dexamethasone [22].

A recent analysis observed an increase in free fatty acids in the colonic tissue of mice with acute colitis and during early repair phases. VLCFAs were enriched, whereas the levels of short- and long-chain fatty acids did not change. This study showed a marked increase in free lignoceric acid and nervonic acid in the injured epithelial cells [23]. VLCFAs were released from the injured intestine and activated peroxisome proliferator-activated receptor (PPAR) gamma in intestinal stem cells to accelerate the repair of intestinal epithelia. This experimental study showed that compared to other fatty acids, VLCFAs, in particular, are enriched in the acutely injured intestine and also during early repair [23].

In the IL-10 knock-out mouse, which develops severe IBD, the increase in saturated, very long-chain ceramides in macrophages is critical for the higher expression of inflammatory genes. This study showed a decrease in most sphingomyelins and a concomitant rise in ceramides in IL-10-null macrophages [24]. Inappropriate de novo synthesis of monounsaturated fatty acids was identified as the cause of the accumulation of VLCFAs. Blocking the synthesis of very long-chain ceramides, as well as oral intake of monounsaturated fatty acids, improved colonic inflammation in these animals [24].

However, in the feces of patients with IBD, levels of sphingolipids like sphingomyelin were elevated compared to healthy individuals [25].

Experimental studies indicate a role for VLCFAs in IBD pathology and diagnosis [23,24]. However, current research on the role of VLCFAs in human IBD is limited and there is a gap in our understanding of the role of VLCFAs. To our knowledge, levels of VLCFAs in the fecal samples of patients and their association with IBD severity have so far not been investigated. Our study aims to examine fecal levels of nervonic acid, lignoceric acid, and pentacosanoic acid, to identify associations with clinical markers of IBD severity.

## 2. Materials and Methods

### 2.1. Patients

Patients aged 18 years or older with IBD were randomly selected from the tertiary care center outpatient or inpatient clinic. Recruitment for the study took place from 6 December 2021 to 31 January 2023. Diagnosis of IBD was established using histologic, endoscopic, and clinical criteria [26]. Pregnant women and individuals with coagulopathy were excluded from the study. Patients with primary sclerosing cholangitis were also excluded.

### 2.2. Bristol Stool Chart

Stools were classified according to the Bristol stool chart as follows: type 1 and 2 indicating constipation (5 patients), type 3 and 4 indicating normal stool (15 patients), type 5 and 6 indicating diarrhea (33 patients), and type 7 indicating watery stool (9 patients).

### 2.3. Gastrointestinal Symptom Rating Scale

The Gastrointestinal Symptom Rating Scale (GSRS) is a questionnaire used to assess gastrointestinal symptoms [27]. It consists of questions to rate various symptoms, commonly associated with gastrointestinal disorders, and covers symptoms such as abdominal pain, bloating, diarrhea, constipation, and general discomfort related to digestion. Two patients with IBD had very severe symptoms, 36 patients had moderate symptoms, 21 patients had mild symptoms, and one patient had no symptoms. The score of two patients was not documented.

### 2.4. Analysis of Fecal Fatty Acids

Stool samples from patients and healthy controls (including hospital staff, students, and patients’ partners) were collected using 70% isopropanol. These fecal samples have been used before to measure fecal bile acids [28]. Samples were stored at −80 °C until further processing by using the gentleMACS^TM^ Dissociator (Miltenyi Biotec GmbH, Bergisch Gladbach, Germany) for homogenization. To determine the dry weight, 1.0 mL of the homogenized mixture was dried in a vacuum centrifuge. The homogenates were then diluted to a final concentration of 2.0 mg dry weight/mL for further examination. The quantification of fecal fatty acids was performed using gas chromatography coupled with mass spectrometry (GC-MS) after the derivatization of fatty acid methyl ester, as described previously [29], with some modifications. In brief, the initial column temperature of 50 °C was held for 0.75 min, increased with 40 °C/min to 110 °C, with 6 °C/min to 210 °C, with 15 °C/min to 250 °C and held for 2 min.

A calibration curve for all measured fatty acids, based on selected ion monitoring and 19-methyleicosanoate as an internal standard, was used. This approach allows quantification via GC-MS. The details of the quantification and the method validation are described in Ecker et al. [29]. In the current study, data were calculated for nervonic acid together with the data of the VLCFAs lignoceric acid and pentacosanoic acid.

### 2.5. Analysis of C-Reactive Protein

The evaluation of C-reactive protein (CRP) levels was conducted using an improved approach for immunoturbidimetric assays. A Cobas Pro analyzer and matching Roche assays (Penzberg, Germany) were used for these tests.

### 2.6. Analysis of Creatinine and Calculation of Glomerular Filtration Rate

Creatinine was converted to glycine, formaldehyde, and hydrogen peroxide by creatininase, creatinase, and sarcosine oxidase as part of the enzymatic method for measuring serum creatinine. Using 4-aminophenazone and HTIBa as substrates, peroxidase uses the liberated hydrogen peroxide to create a quinoneiminine dye. The concentration of creatinine in the reaction mixture directly correlates with the quinoneiminine dye’s color intensity. A Cobas Pro analyzer and matching assays from Roche were used for this test. The formula outlined by Levey et al. [30] was applied to the glomerular filtration rate calculation.

### 2.7. Analysis of Fecal Calprotectin

The measurement of fecal calprotectin was conducted with the Quanta Flash Calprotectin reagent (Inova Diagnostics, San Diego, CA, USA). In everyday clinical practice, fecal calprotectin stands out as the key non-invasive indicator for assessing IBD activity, aligning closely with endoscopic observations [31,32,33,34].

### 2.8. Statistical Analysis

Data are depicted as boxplots. Each box represents the interquartile range (IQR), with the bottom and top edges corresponding to the first quartile (Q1) and third quartile (Q3), respectively. The line inside the box represents the median (50th percentile) of the data. Outliers in boxplots are indicated by circles or asterisks. The statistical methods employed included the Mann–Whitney U-test, the Kruskal–Wallis test, receiver operating characteristic curve analysis, multiple linear regression, and Spearman correlation (SPSS Statistics 26.0 program, IBM, Leibniz Rechenzentrum, München, Germany). The tables present data in terms of median, minimum, and maximum values. Statistical significance was defined as a *p*-value < 0.05.

## 3. Results

### 3.1. Fecal Fatty Acids in Patients with Inflammatory Bowel Disease and Healthy Controls

The study cohort consisted of 62 patients with IBD, comprising 38 patients with Crohn’s disease (CD) and 24 patients with ulcerative colitis (UC), alongside 17 healthy controls (Table 1). The sex distribution and age of the patients and controls were similar (Table 1).

Nervonic acid (FA 24:1), and for comparison, the VLCFAs lignoceric acid (FA 24:0) as well as pentacosanoic acid (FA 25:0), were measured in the feces of patients with IBD and healthy controls. Fecal levels of all three fatty acids did not differ between male and female controls (*p* > 0.05 for all). Lignoceric acid (r = −0.726, *p* = 0.001) and pentacosanoic acid (r = −0.726, *p* = 0.001) were negatively correlated with age in healthy controls, while nervonic acid (r = 0.247, *p* = 0.356) showed no significant correlation in this group. In patients with IBD, fecal levels of nervonic acid, lignoceric acid, and pentacosanoic acid did not differ between females and males and showed no correlation with age or body mass index (BMI) (*p* > 0.05 for all).

When stratified by age, lignoceric acid, nervonic acid, and pentacosanoic acid did not change with increasing age (Table 2). Body mass index, C-reactive protein, creatinine, and fecal calprotectin levels were similar between these groups (Table 2). As expected, the glomerular filtration rate declined with increasing age (Table 2) [35].

Comparing patients with CD and UC, the levels of the analyzed fatty acids showed no significant differences (*p* = 0.908 for lignoceric acid, *p* = 0.665 for nervonic acid, and *p* = 0.269 for pentacosanoic acid).

**Figure 1:**
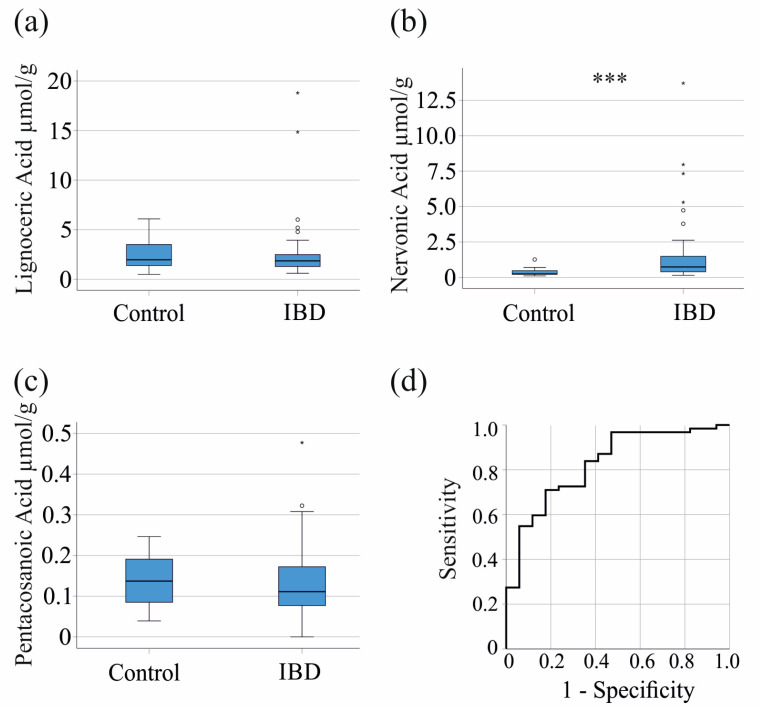
Fecal nervonic acid is increased in patients with IBD. Comparison of fecal very-long chain fatty acid levels between healthy controls and patients with IBD. (**a**) Concentration of lignoceric acid; (**b**) nervonic acid; and (**c**) pentacosanoic acid in the stool of healthy controls and patients with IBD; (**d**) receiver operating characteristic curve for the discrimination of patients and controls by fecal nervonic acid levels. *** *p* < 0.001.

Lignoceric acid (*p* = 0.756) and pentacosanoic acid (*p* = 0.655) showed no significant differences between patients with IBD and healthy controls. Of clinical relevance, we identified significantly higher fecal levels of nervonic acid in patients with IBD (*p* < 0.001) (Figure 1a–c). A receiver operating characteristic (ROC) curve analysis (Figure 1d) revealed that nervonic acid at a concentration of 0.49 µmol/g discriminated IBD patients from healthy controls with a sensitivity of 71% and a specificity of 82% (AUROC = 0.827, *p* < 0.001). These data show that fecal nervonic acid is specifically elevated in IBD.

### 3.2. Fecal Nervonic Acid Levels Correlate with Clinical Markers of Inflammation in Inflammatory Bowel Disease

We aimed to analyze whether increased levels of nervonic acid are associated with disease activity or inflammation in patients with IBD by performing correlation analyses with clinically established markers of inflammation. Lignoceric acid and pentacosanoic acid were not associated with serum CRP, fecal calprotectin, and parameters of renal function (Table 3). Of note, nervonic acid positively correlated with serum CRP, fecal calprotectin, and glomerular filtration rate in the IBD cohort (Table 3).

**Figure 2:**
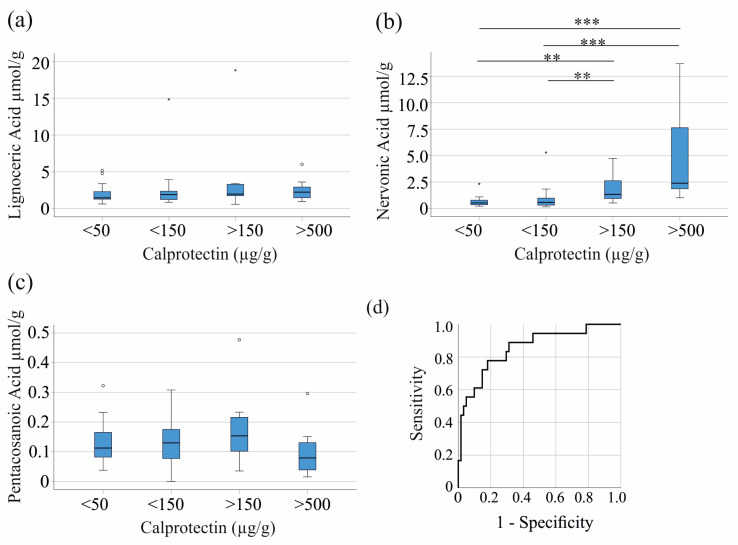
Relation of fecal fatty acids and fecal calprotectin. (**a**) Concentrations of lignoceric acid; (**b**) nervonic acid; and (**c**) pentacosanoic acid in the stool of IBD patients with fecal calprotectin levels <50 µg/g (twenty-five patients), <150 µg/g (twenty patients), >150 µg/g (eight patients), and >500 µg/g (eight patients); (**d**) receiver operating characteristic curve for the discrimination of patients with fecal calprotectin < and ≥120 µg/g by fecal nervonic acid levels. ** *p* < 0.01, *** *p* < 0.001.

To further clarify the relationship between fecal fatty acids and fecal calprotectin, we analyzed the fecal concentration of VLCFAs in relation to fecal calprotectin levels. As indicated by the Spearman correlation coefficients (Table 3), lignoceric acid and pentacosanoic acid remained unchanged regardless of the respective fecal calprotectin levels (Figure 2a,c). Notably, fecal levels of nervonic acid increased with higher fecal calprotectin (Figure 2b). In our cohort, twenty-five patients had fecal calprotectin levels below 50 µg/g, twenty patients had levels between 50 and 150 µg/g, eight patients had levels between 150 and 500 µg/g, and eight patients had levels above 500 µg/g. Data for one patient were not documented.

Fecal calprotectin levels ≥ 120 µg/g are considered positive by the assay used in our study. A receiver operating characteristic (ROC) curve analysis shows that fecal nervonic acid at a concentration of 0.94 µmol/g discriminates IBD patients with calprotectin levels < and ≥ 120 µg/g with a sensitivity of 78% and a specificity of 82% (area under the receiver operating curve = 0.856, *p* < 0.001) (Figure 2d). Taken together, these results indicate that fecal nervonic acid may be of value for diagnosing IBD and monitoring disease activity.

Multiple regression analysis using fecal nervonic acid as the dependent variable, and fecal calprotectin, age, and sex as independent variables revealed that these variables were able to predict fecal nervonic acid levels, F(3,57)18.25, *p* < 0.001. Here, only the effect of fecal calprotectin was significant (*p* < 0.001); age (*p* = 0.210) and sex (*p* = 0.958) were not significantly associated with fecal calprotectin levels.

### 3.3. Relation of Fecal Fatty Acids with Stool Consistency and Gastrointestinal Symptom Rating Scale

**Figure 3:**
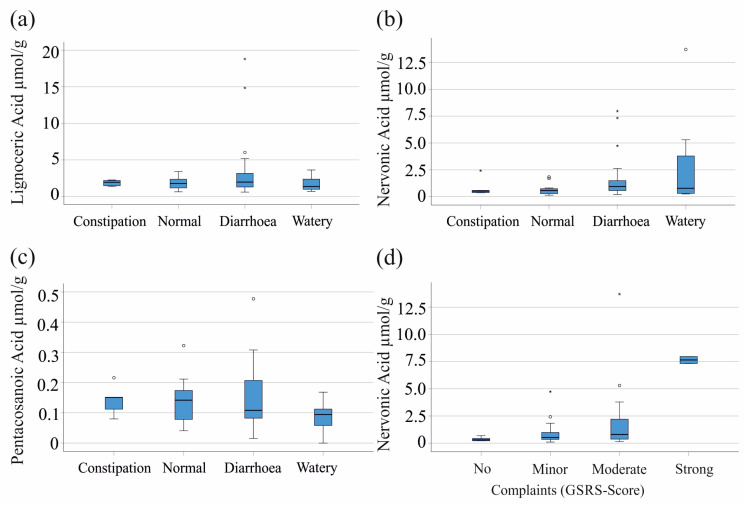
Association of fecal fatty acids with stool consistency and the Gastrointestinal Symptom Rating Scale (GSRS). (**a**) Concentrations of lignoceric acid; (**b**) nervonic acid; and (**c**) pentacosanoic acid in patients with constipation, normal stool, diarrhea, and watery stool; (**d**) fecal nervonic acid in patients with increasing GSRS scores.

In addition to laboratory parameters, we further evaluated whether levels of fecal fatty acids were associated with stool consistency, which was documented by the patients using the Bristol stool chart. Fecal levels of lignoceric acid (p = 0.638) and pentacosanoic acid (*p* = 0.287) did not show significant associations with the Bristol stool score (Figure 3a,c). Notably, patients with higher Bristol stool scores (types 5–7) consistently showed a non-significant increase in nervonic acid levels (*p* = 0.091) (Figure 3b).

The Gastrointestinal Symptom Rating Scale (GSRS) is a clinical tool used to assess gastrointestinal symptoms and their severity. In our cohort, the GSRS was not associated with fecal lignoceric (*p* = 0.537) and pentacosanoic acid (*p* = 0.121). Noteworthy, fecal nervonic acid was highest in the two patients with IBD, with very strong complaints in comparison to those with no, minor, and moderate complaints (Figure 3d). The analysis of other inflammatory markers revealed that fecal calprotectin increased with higher GSRS (*p* = 0.014), while serum CRP did not significantly change (*p* = 0.096).

### 3.4. Effects of Medication on Fecal Fatty Acid Composition

**Figure 4:**
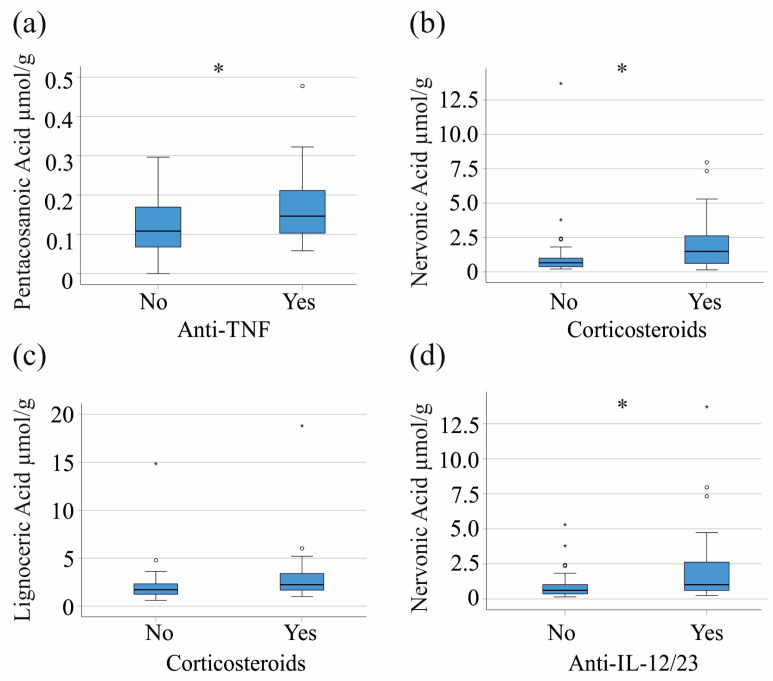
Impact of IBD therapy on fecal fatty acids. Associations of fecal fatty acids with anti-TNF, corticosteroid and anti-IL-12/23 therapy. (**a**) Fecal pentacosanoic acid levels of patients treated or not treated with anti-TNF antibodies; (**b**) fecal nervonic acid of patients treated or not treated with corticosteroids; (**c**) fecal lignoceric acid of patients treated or not treated with corticosteroids; (**d**) fecal nervonic acid of patients treated or not treated with anti-IL-12/23 antibodies. * *p* < 0.05.

To assess whether and how therapy affects fecal fatty acid content, we analyzed the impact of various treatment modalities on fecal fatty acid composition and inflammatory markers in our IBD cohort. The 22 patients who were treated with a chimeric monoclonal antibody against tumor necrosis factor (TNF) alpha [36] had higher fecal pentacosanoic acid levels compared to those who did not receive anti-TNF therapy (*p* = 0.046) (Figure 4a), with no significant differences in CRP and fecal calprotectin between these groups (*p* > 0.05 for both). Corticosteroids were administered to 17 IBD patients, with those treated exhibiting higher fecal levels of nervonic acid (*p* = 0.022) and lignoceric acid (*p* = 0.064) (Figure 4b,c). The fecal calprotectin (*p* = 0.182) and serum CRP (*p* = 0.208) of patients receiving corticosteroids were not increased. Mesalazine (21 patients) and azathioprine (6 patients) were not associated with altered fecal fatty acid levels. Anti-interleukin (IL)-12/23 antibody therapy (18 patients) was related to higher nervonic acid (*p* = 0.014) (Figure 4d), but not with altered serum CRP (*p* = 0.114) or fecal calprotectin (*p* = 0.114).

## 4. Discussion

The present study is the first to show that fecal nervonic acid levels increase proportionally with disease activity in patients with IBD.

Nervonic acid at a concentration of 0.49 µmol/g discriminated patients from controls with a sensitivity of 71% and a specificity of 82%. In comparison, the sensitivity of fecal calprotectin in detecting IBD at a threshold of 50 µg/g was 93%, and the specificity was 62% in a clinical setting [37]. Thus, fecal calprotectin has higher sensitivity, while fecal nervonic acid shows superior specificity. Therefore, measuring nervonic acid in addition to fecal calprotectin may improve the diagnostic performance for IBD.

Fecal calprotectin is an established biomarker of bowel inflammation independent of the underlying disease [31,32], and further studies are required to assess whether fecal nervonic acid levels are specifically increased in IBD.

Our study evaluated levels of the VLCFAs nervonic acid, lignoceric acid, and pentacosanoic acid in the feces of patients with IBD. To compare fecal lipid levels between individuals, accurate normalization of the samples was required. Sample wet weight, stool dry weight, and fecal protein concentration were used to normalize lipid levels. Patients with IBD may have loose, watery stools [38], so in our analysis, fatty acid levels were normalized to stool dry weight. The comparable levels of the analyzed fatty acids in the feces of patients with varying stool consistency indicate that this normalization method effectively adjusts for variability in stool consistency.

Patients with a high GSRS score who also had elevated calprotectin levels displayed a marked increase in fecal nervonic acid levels. Otherwise, fecal VLCFA levels did not change significantly with symptoms. This clearly demonstrates that higher levels of fecal nervonic acid in patients with active IBD are a marker of disease activity and not disease symptoms or stool consistency.

Nervonic acid has been shown to be acetylated to sphingolipids, including sphingomyelin and ceramide [20,39]. Host-derived sphingolipids are key metabolic markers of IBD, showing a high increase in samples from patients with both UC and CD [25,40]. In patients with UC, fecal sphingomyelin levels were about 10 times higher during active disease than during remission [40]. Moreover, this study demonstrated that distinct sphingomyelin species were elevated in CD and UC patients compared to healthy controls, whereas ceramide species were specifically increased in CD. The authors speculate that this increase in sphingomyelins may serve as a compensatory mechanism for the deficiency of bacterially produced sphingolipids [40].

Higher levels of fecal nervonic acid may indicate an increased production of specific sphingomyelin species in IBD. Thus, one could hypothesize that high levels of nervonic acid reflect the body’s attempt to counteract inflammation in IBD. However, our observational study cannot elucidate the pathways responsible for elevated levels of fecal nervonic acid while other fatty acids of similar length remain normal.

The oxidation of VLCFA takes place in peroxisomes, whereas fatty acids with fewer carbon atoms are oxidized in mitochondria [41]. The number of peroxisomes in intestinal epithelial cells of patients with CD was found to be reduced compared to controls and further decreased with increasing inflammation [42]. In murine colitis, peroxisomes within crypts were increased during the acute phase and early repair phase and decreased during the late repair phase [23]. Reduced peroxisomal oxidation of VLCFA may contribute to higher levels in feces but cannot explain the selective increase in nervonic acid. One analysis has shown that peroxisome-deficient cells handle monounsaturated and saturated fatty acids differently [43], but this needs further study.

The absorption of dietary VLCFAs is largely mediated by intestinal CD36 [44], and the number of CD36-expressing cells is reduced in the inflamed mucosa of patients with IBD [45], but impaired intestinal uptake of VLCFA will affect the levels of all VLCFAs. Thus, the intestinal absorption and oxidation of VLCFA cannot explain the selective increase in fecal nervonic acid levels.

Patients with IBD have been found to have similar [46] and lower daily intakes of various fats [47]. Fish oils, which are rich in nervonic acid, also contain lignoceric acid, and it remains to be investigated whether diet plays a role in higher fecal nervonic acid levels [48].

In colonic tissues from mice with acute colitis and during early repair phases, accumulation of VLCFAs and significant increases in lignoceric and nervonic acids were observed in injured epithelial cells [23]. In patients with IBD, fecal nervonic acid was specifically induced. The acute colitis in the mouse model may differ from the chronic colitis seen in most of our patients, and studies of chronic colitis in mice may resolve this discrepancy. Increased production and release of nervonic acid in the intestinal cells of patients with IBD is currently the most plausible explanation for the higher levels in feces.

Nervonic acid was shown to reverse the accumulation of saturated VLCFAs and to protect against their cytotoxic effects [49]. Thus, elevated fecal nervonic acid levels in IBD may contribute to the normalization of other saturated VLCFA levels and may be considered protective.

Corticosteroids are potent anti-inflammatory drugs that also have adverse effects, such as lipid abnormalities and diabetogenic metabolic states [50,51]. Patients on corticosteroid therapy had higher fecal levels of nervonic acid than patients not treated with these drugs. Fecal nervonic acid levels of patients receiving interleukin 12/23 antibodies were also higher than those treated with other drugs. Serum CRP and fecal calprotectin levels were similar between these groups. The observed increase in the fecal pentacosanoic acid in patients treated with TNF antibodies was modest and requires confirmation.

To our knowledge, this is the first study to investigate associations between fecal VLCFA and fecal calprotectin. A limitation is the collection of only a single fecal sample and the lack of sphingolipid quantification in patients’ stools. This analysis cannot distinguish between free and bound VLCFAs. This is a descriptive study that is not suitable for understanding cause-and-effect relationships. Although the study is descriptive and does not provide explanations for the observed elevated fecal nervonic acid levels, it identifies nervonic acid as a potential novel biomarker in IBD.

## 5. Conclusions

This analysis showed that the monounsaturated VLCFA nervonic acid but not saturated VLCFAs are higher in the feces of patients with IBD compared to healthy controls. Elevated fecal nervonic acid levels in IBD positively correlate with fecal calprotectin levels. Defined concentrations of fecal nervonic acid can distinguish patients with IBD from healthy controls. Thus, fecal nervonic acid is a promising biomarker for diagnosing IBD and monitoring disease activity and treatment response. Measuring nervonic acid in addition to fecal calprotectin may improve the diagnostic performance for IBD.

## Figures and Tables

**Table 1:** Characteristics of the patient and control cohort.

Characteristics	IBD	Controls
Number (female/male)	62 (28/34)	17 (10/7)
Age (years)	42 (19–78)	48 (23–78)
Body Mass Index (kg/m^2^)	24 (16–44)	not determined
C-reactive protein (mg/L)	3 (0–144)	not determined
Creatinine (mg/dL)	0.85 (0.51–1.25)	not determined
Glomerular filtration rate (mL/min)	99 (61–136)	not determined
Fecal calprotectin (µg/g)	62 (17–1616)	not determined

**Table 2:** Characteristics of the patients with IBD stratified by age.

Characteristics						
Age (years)	20–30	31–40	41–50	51–60	>61	*p*-value
Number (female/male)	14 (6/8)	14 (8/6)	9 (2/7)	18 (10/8)	7(2/5)	0.360
Lignoceric acid µmol/g	1.95 (0.60–3.62)	1.47 (0.64–6.02)	1.86 (1.00–14.84)	1.67 (0.69–18.80)	2.24 (1.14–3.40)	0.827
Nervonic acid µmol/g	1.42 (0.27–13.70)	0.56 (0.24–2.07)	0.77 (0.27–3.78)	0.61 (0.10–2.62)	0.68 (0.25–7.32)	0.250
Pentacosanoic acid µmol/g	0.11 (0–0.22)	0.10 (0.04–0.31)	0.13 (0.06–0.25)	0.10 (0.04–0.48)	0.15 (0.05–0.32)	0.544
Body Mass Index (kg/m^2^)	23 (16–28)	26 (17–35)	23 (20–25)	24 (21–44)	24 (18–35)	0.553
C-reactive protein (mg/L)	10 (1–57)	4 (0–18)	2 (1–11)	1 (0–144)	2 (1–55)	0.264
Creatinine (mg/dL)	0.83 (0.51–1.12)	0.82 (0.59–0.99)	0.83 (0.76–1.02)	0.85 (0.70–1.06)	0.89 (0.74–1.25)	0.389
Glomerular filtration rate (mL/min)	112 (91–136)	112 (84–122)	106 (87–110)	92 (62–103)	83 (61–97)	<0.001
Fecal calprotectin (µg/g)	121 (17–1616)	65 (17–639)	55 (33–538)	45 (18–1543)	34 (18–883)	0.126

**Table 3:** Spearman correlation coefficients for the correlations of fecal fatty acids with C-reactive protein, fecal calprotectin, creatinine, and glomerular filtration rate in patients with IBD. * *p* < 0.05, ** *p* < 0.01, *** *p* < 0.001.

	Lignoceric Acid	Nervonic Acid	Pentacosanoic Acid
C-reactive protein	0.042	0.376 **	−0.217
Fecal Calprotectin	0.175	0.575 ***	−0.017
Creatinine	−0.035	−0.171	0.080
Glomerular filtration rate	−0.021	0.267 *	−0.173

## Data Availability

Original data are attached as Supplementary Materials and can also be obtained by the corresponding author on request.

## Supplementary Materials

The following supporting information can be downloaded at: https://www.mdpi.com/article/10.3390/biomedicines12122764/s1.

## References

[1] Torres J., Mehandru S., Colombel J.F., Peyrin-Biroulet L. (2017). Crohn’s disease. Lancet.

[2] Le Berre C., Honap S., Peyrin-Biroulet L. (2023). Ulcerative colitis. Lancet.

[3] Kaplan G.G., Windsor J.W. (2021). The four epidemiological stages in the global evolution of inflammatory bowel disease. Nat. Rev. Gastroenterol. Hepatol..

[4] De Souza H.S., Fiocchi C. (2016). Immunopathogenesis of IBD: Current state of the art. Nat. Rev. Gastroenterol. Hepatol..

[5] Kunst C., Schmid S., Michalski M., Tumen D., Buttenschon J., Muller M., Gulow K. (2023). The Influence of Gut Microbiota on Oxidative Stress and the Immune System. Biomedicines.

[6] Obermeier F., Hofmann C., Falk W. (2010). Inflammatory bowel diseases: When natural friends turn into enemies-the importance of CpG motifs of bacterial DNA in intestinal homeostasis and chronic intestinal inflammation. Int. J. Inflam..

[7] Di Sabatino A., Ciccocioppo R., Luinetti O., Ricevuti L., Morera R., Cifone M.G., Solcia E., Corazza G.R. (2003). Increased enterocyte apoptosis in inflamed areas of Crohn’s disease. Dis. Colon. Rectum.

[8] Hagiwara C., Tanaka M., Kudo H. (2002). Increase in colorectal epithelial apoptotic cells in patients with ulcerative colitis ultimately requiring surgery. J. Gastroenterol. Hepatol..

[9] Marini M., Bamias G., Rivera-Nieves J., Moskaluk C.A., Hoang S.B., Ross W.G., Pizarro T.T., Cominelli F. (2003). TNF-alpha neutralization ameliorates the severity of murine Crohn’s-like ileitis by abrogation of intestinal epithelial cell apoptosis. Proc. Natl. Acad. Sci. USA.

[10] Patankar J.V., Becker C. (2020). Cell death in the gut epithelium and implications for chronic inflammation. Nat. Rev. Gastroenterol. Hepatol..

[11] Strater J., Wellisch I., Riedl S., Walczak H., Koretz K., Tandara A., Krammer P.H., Moller P. (1997). CD95 (APO-1/Fas)-mediated apoptosis in colon epithelial cells: A possible role in ulcerative colitis. Gastroenterology.

[12] Calder P.C. (2002). Dietary modification of inflammation with lipids. Proc. Nutr. Soc..

[13] Piotrowska M., Binienda A., Fichna J. (2021). The role of fatty acids in Crohn’s disease pathophysiology—An overview. Mol. Cell Endocrinol..

[14] Seki H., Tani Y., Arita M. (2009). Omega-3 PUFA derived anti-inflammatory lipid mediator resolvin E1. Prostaglandins Other Lipid Mediat..

[15] Yan D., Ye S., He Y., Wang S., Xiao Y., Xiang X., Deng M., Luo W., Chen X., Wang X. (2023). Fatty acids and lipid mediators in inflammatory bowel disease: From mechanism to treatment. Front. Immunol..

[16] Scaioli E., Sartini A., Bellanova M., Campieri M., Festi D., Bazzoli F., Belluzzi A. (2018). Eicosapentaenoic Acid Reduces Fecal Levels of Calprotectin and Prevents Relapse in Patients With Ulcerative Colitis. Clin. Gastroenterol. Hepatol..

[17] Alhouayek M., Ameraoui H., Muccioli G.G. (2021). Bioactive lipids in inflammatory bowel diseases—From pathophysiological alterations to therapeutic opportunities. Biochim. Biophys. Acta Mol. Cell Biol. Lipids.

[18] Espinoza K.S., Snider A.J. (2024). Therapeutic Potential for Sphingolipids in Inflammatory Bowel Disease and Colorectal Cancer. Cancers.

[19] Keppley L.J.W., Walker S.J., Gademsey A.N., Smith J.P., Keller S.R., Kester M., Fox T.E. (2020). Nervonic acid limits weight gain in a mouse model of diet-induced obesity. FASEB J..

[20] Namiecinska M., Piatek P., Lewkowicz P. (2024). Nervonic Acid Synthesis Substrates as Essential Components in Profiled Lipid Supplementation for More Effective Central Nervous System Regeneration. Int. J. Mol. Sci..

[21] Phung N.V., Rong F., Xia W.Y., Fan Y., Li X.Y., Wang S.A., Li F.L. (2023). Nervonic acid and its sphingolipids: Biological functions and potential food applications. Crit. Rev. Food Sci. Nutr..

[22] Yuan S.N., Wang M.X., Han J.L., Feng C.Y., Wang M., Wang M., Sun J.Y., Li N.Y., Simal-Gandara J., Liu C. (2023). Improved colonic inflammation by nervonic acid via inhibition of NF-kappaB signaling pathway of DSS-induced colitis mice. Phytomedicine.

[23] Guo X., Zhou J., La Y., Liu X., Yuan Y., Ye J., Zhang Z., Chen H., Ma Y., Zhong Z. (2024). Very long-chain fatty acids control peroxisome dynamics via a feedback loop in intestinal stem cells during gut regeneration. Dev. Cell.

[24] York A.G., Skadow M.H., Oh J., Qu R., Zhou Q.D., Hsieh W.Y., Mowel W.K., Brewer J.R., Kaffe E., Williams K.J. (2024). IL-10 constrains sphingolipid metabolism to limit inflammation. Nature.

[25] Franzosa E.A., Sirota-Madi A., Avila-Pacheco J., Fornelos N., Haiser H.J., Reinker S., Vatanen T., Hall A.B., Mallick H., McIver L.J. (2019). Gut microbiome structure and metabolic activity in inflammatory bowel disease. Nat. Microbiol..

[26] Sturm A., Maaser C., Calabrese E., Annese V., Fiorino G., Kucharzik T., Vavricka S.R., Verstockt B., van Rheenen P., Tolan D. (2019). ECCO-ESGAR Guideline for Diagnostic Assessment in IBD Part 2: IBD scores and general principles and technical aspects. J. Crohns Colitis.

[27] Schafer S.K., Weidner K.J., Hoppner J., Becker N., Friedrich D., Stokes C.S., Lammert F., Kollner V. (2017). Design and validation of a German version of the GSRS-IBS—An analysis of its psychometric quality and factorial structure. BMC Gastroenterol..

[28] Sommersberger S., Gunawan S., Elger T., Fererberger T., Loibl J., Huss M., Kandulski A., Krautbauer S., Muller M., Liebisch G. (2023). Altered fecal bile acid composition in active ulcerative colitis. Lipids Health Dis..

[29] Ecker J., Scherer M., Schmitz G., Liebisch G. (2012). A rapid GC-MS method for quantification of positional and geometric isomers of fatty acid methyl esters. J. Chromatogr. B.

[30] Levey A.S., Stevens L.A., Schmid C.H., Zhang Y.L., Castro A.F., Feldman H.I., Kusek J.W., Eggers P., Van Lente F., Greene T. (2009). A new equation to estimate glomerular filtration rate. Ann. Intern. Med..

[31] Alghoul Z., Yang C., Merlin D. (2022). The Current Status of Molecular Biomarkers for Inflammatory Bowel Disease. Biomedicines.

[32] Jukic A., Bakiri L., Wagner E.F., Tilg H., Adolph T.E. (2021). Calprotectin: From biomarker to biological function. Gut.

[33] Rogler G., Aldeguer X., Kruis W., Lasson A., Mittmann U., Nally K., Peyrin-Biroulet L., Schoepfer A., Vatn M., Vavricka S. (2013). Concept for a rapid point-of-care calprotectin diagnostic test for diagnosis and disease activity monitoring in patients with inflammatory bowel disease: Expert clinical opinion. J. Crohns Colitis.

[34] Ukashi O., Kopylov U., Ungar B., Talan Asher A., Shachar E., Engel T., Albshesh A., Yablecovitch D., Lahat A., Eliakim R. (2024). Fecal calprotectin diagnostic level gradient along the small bowel in patients with Crohn’s disease. J. Crohns Colitis.

[35] Glassock R.J., Winearls C. (2009). Ageing and the glomerular filtration rate: Truths and consequences. Trans. Am. Clin. Climatol. Assoc..

[36] Brown S.J., Mayer L. (2007). The immune response in inflammatory bowel disease. Am. J. Gastroenterol..

[37] Freeman K., Taylor-Phillips S., Willis B.H., Ryan R., Clarke A. (2021). Test accuracy of faecal calprotectin for inflammatory bowel disease in UK primary care: A retrospective cohort study of the IMRD-UK data. BMJ Open.

[38] Anbazhagan A.N., Priyamvada S., Alrefai W.A., Dudeja P.K. (2018). Pathophysiology of IBD associated diarrhea. Tissue Barriers.

[39] Fox T.E., Bewley M.C., Unrath K.A., Pedersen M.M., Anderson R.E., Jung D.Y., Jefferson L.S., Kim J.K., Bronson S.K., Flanagan J.M. (2011). Circulating sphingolipid biomarkers in models of type 1 diabetes. J. Lipid Res..

[40] Brown E.M., Ke X., Hitchcock D., Jeanfavre S., Avila-Pacheco J., Nakata T., Arthur T.D., Fornelos N., Heim C., Franzosa E.A. (2019). Bacteroides-Derived Sphingolipids Are Critical for Maintaining Intestinal Homeostasis and Symbiosis. Cell Host Microbe.

[41] Demarquoy J., Le Borgne F. (2015). Crosstalk between mitochondria and peroxisomes. World J. Biol. Chem..

[42] Pinelli M., Makdissi S., Scur M., Parsons B.D., Baker K., Otley A., MacIntyre B., Nguyen H.D., Kim P.K., Stadnyk A.W. (2024). Peroxisomal cholesterol metabolism regulates yap-signaling, which maintains intestinal epithelial barrier function and is altered in Crohn’s disease. Cell Death Dis..

[43] Ali H., Morito K., Hasi R.Y., Aihara M., Hayashi J., Kawakami R., Kanemaru K., Tsuchiya K., Sango K., Tanaka T. (2022). Characterization of uptake and metabolism of very long-chain fatty acids in peroxisome-deficient CHO cells. Biochim. Biophys. Acta Mol. Cell Biol. Lipids.

[44] Drover V.A., Nguyen D.V., Bastie C.C., Darlington Y.F., Abumrad N.A., Pessin J.E., London E., Sahoo D., Phillips M.C. (2008). CD36 mediates both cellular uptake of very long chain fatty acids and their intestinal absorption in mice. J. Biol. Chem..

[45] Ortiz-Masia D., Diez I., Calatayud S., Hernandez C., Cosin-Roger J., Hinojosa J., Esplugues J.V., Barrachina M.D. (2012). Induction of CD36 and thrombospondin-1 in macrophages by hypoxia-inducible factor 1 and its relevance in the inflammatory process. PLoS ONE.

[46] Peters V., Tigchelaar-Feenstra E.F., Imhann F., Dekens J.A.M., Swertz M.A., Franke L.H., Wijmenga C., Weersma R.K., Alizadeh B.Z., Dijkstra G. (2021). Habitual dietary intake of IBD patients differs from population controls: A case-control study. Eur. J. Nutr..

[47] Opstelten J.L., de Vries J.H.M., Wools A., Siersema P.D., Oldenburg B., Witteman B.J.M. (2019). Dietary intake of patients with inflammatory bowel disease: A comparison with individuals from a general population and associations with relapse. Clin. Nutr..

[48] Suseno S.H., Nurjanah N., Yoshiara Y., Saraswati S. (2013). Determination of extraction temperature and period of fish oil from tilapia (*Oreochromis niloticus*) by product using wet rendering method. KnE Life Sci..

[49] Terluk M.R., Tieu J., Sahasrabudhe S.A., Moser A., Watkins P.A., Raymond G.V., Kartha R.V. (2022). Nervonic Acid Attenuates Accumulation of Very Long-Chain Fatty Acids and is a Potential Therapy for Adrenoleukodystrophy. Neurotherapeutics.

[50] Bruscoli S., Febo M., Riccardi C., Migliorati G. (2021). Glucocorticoid Therapy in Inflammatory Bowel Disease: Mechanisms and Clinical Practice. Front. Immunol..

[51] Liu D., Ahmet A., Ward L., Krishnamoorthy P., Mandelcorn E.D., Leigh R., Brown J.P., Cohen A., Kim H. (2013). A practical guide to the monitoring and management of the complications of systemic corticosteroid therapy. Allergy Asthma Clin. Immunol..

