# Supplementary material for: Fecal Nervonic Acid as a Biomarker for Diagnosing and Monitoring Inflammatory Bowel Disease
Source: Biomedicines. 2024 Dec 4;12(12):2764. doi: 10.3390/biomedicines12122764 (PMC11673069; doi:10.3390/biomedicines12122764)
Supplement: Supplementary file 1 [file biomedicines-12-02764-s001.zip › biomedicines-3340624-supplementary.pdf]

	Patient/Control	Calprotectin mg/dl	Lignoceric acid µmol/g	Nervonic Acid µmol/g	Pentacosanoic Acid µmol/g
Male	CD	25	1,43	0,82	0,08
Female	CD	77	1,47	0,48	0,09
Female	CD	304	1,98	4,73	0,10
Male	CD	18	2,48	1,10	0,23
Female	CD	75	1,23	0,72	0,07
Male	CD	251	2,17	1,81	0,15
Female	CD	38	3,16	0,80	0,17
Male	CD	35	1,76	0,61	0,10
Male	CD	265	0,60	0,72	0,04
Male	CD	35	4,78	0,77	0,21
Female	CD	77	1,96	0,57	0,11
Male	CD	29	2,31	0,98	0,19
Female	CD	58	2,33	0,50	0,17
Female	CD	170	18,80	2,62	0,48
Female	CD	44	1,85	2,33	0,12
Female	CD	175	3,41	1,34	0,23
Male	CD	347	3,31	3,78	0,17
Female	CD	24	1,46	0,62	0,08
Male	CD	42	1,86	0,52	0,13
Female	CD	146	1,94	0,52	0,22
Female	CD	32	2,32	0,40	0,19
Male	CD	81	3,42	1,70	0,21
Female	CD	639	6,02	2,07	0,30
Female	CD	45	1,67	0,39	0,17
Male	CD	1527	2,25	2,42	0,15
Male	CD	538	1,68	1,65	0,11
Female	CD	34	1,06	0,27	0,06
Female	CD	18	1,35	0,31	0,11
Female	CD	55	14,84	0,98	0,25
Male	CD	17	1,47	0,39	0,11
Male	CD	143	1,36	0,25	0,09
Male	CD	143	1,00	0,71	0,06
Male	CD	33	1,29	0,28	0,10
Male	CD	80	1,04	0,41	0,06
Female	CD	24	1,24	1,09	0,04
Female	CD	100	2,18	0,58	0,15
Male	CD	17	0,64	0,24	0,04
Female	CD	62	1,19	0,15	0,07
Female	UC	1097	2,25	7,96	0,02
Male	UC	883	2,24	7,32	0,05
Male	UC	1543	0,96	2,36	0,05
Male	UC	1616	3,62	13,70	0,03
Male	UC	20	3,23	0,58	0,14
Male	UC	805	1,29	1,01	0,11
Male	UC	57	0,98	5,30	0,00
Male	UC	25	5,19	0,35	0,12
Female	UC	20	1,14	0,25	0,12
Female	UC	73	1,79	0,77	0,18

Male	UC	34	3,40	0,68	0,32
Male	UC	191	1,75	0,94	0,10
Male	UC	77	2,37	1,84	0,17
Male	UC	105	3,93	1,49	0,31
Male	UC	57	2,69	0,94	0,23
Female	UC	328	1,24	1,04	0,09
Male	UC		3,82	0,75	0,25
Female	UC	35	0,69	0,78	0,06
Female	UC	95	2,37	0,27	0,10
Male	UC	83	1,86	0,29	0,16
Male	UC	34	1,45	0,50	0,10
Female	UC	34	1,36	0,37	0,08
Male	UC	19	1,11	0,21	0,04
Female	UC	54	0,86	0,32	0,08
Female	Control		3,16	0,44	0,19
Female	Control		4,02	0,28	0,23
Male	Control		1,98	0,21	0,14
Female	Control		3,50	0,20	0,18
Female	Control		3,76	0,70	0,22
Male	Control		6,09	0,24	0,15
Male	Control		3,73	0,48	0,25
Male	Control		1,39	0,59	0,10
Female	Control		1,61	0,31	0,10
Male	Control		3,40	0,21	0,22
Female	Control		0,94	0,10	0,08
Female	Control		1,75	0,21	0,09
Female	Control		0,65	0,22	0,04
Male	Control		0,49	1,26	0,04
Male	Control		1,42	0,48	0,09
Female	Control		1,96	0,17	0,17
Female	Control		0,92	0,22	0,08
